# Drug-Related Deaths Among Young People in a Scottish Region: A Socio-Ecological Autopsy Approach to Understanding the Context of Drug Deaths

**DOI:** 10.1177/14550725251370436

**Published:** 2025-09-09

**Authors:** Aileen O’Gorman, Stephanie Govenden, Frances Matthewson

**Affiliations:** 1 School of Education & Social Sciences, 6413 University of the West of Scotland, Paisley, Scotland, UK; 2 8930 NHS Highland, Inverness, Scotland, UK; 3 Highland Alcohol and Drugs Partnership, Inverness, Scotland, UK

**Keywords:** drug-related deaths, overdose, risk environments, social autopsy, social determinants, social ecology

## Abstract

**Aims:**

Social, structural and systemic factors are critical to understanding drug-related deaths among adults. The relevance of these factors to young people is not known. This study explores the life experience, drug using histories and the interactions of a group of young people with agencies and services prior to their death. Our approach seeks to look beyond the immediate cause of death and identify broader contextual factors that may have contributed to a death through a “whole-life view”.

**Methods:**

The study developed a socio-ecological autopsy approach informed by social autopsy methods and social ecology and risk environment frameworks. Health, social work, police and post-mortem records of the young people were collated and analysed. Summary narratives, chronologies and descriptive statistics were produced using Excel and NVivo.

**Findings:**

Twenty-one deaths were identified; almost all were due to multi-drug toxicity, mainly heroin mixed with other substances. Almost all the young people had reported mental health issues such as anxiety, depression and self-harm, and had experinced at least one recorded overdose before they died. Most grew up in precarity and poverty in deprived areas. In their short lives, most of this cohort of young people experienced multiple adversities in childhood and as young adults, particularly in the year preceding their death.

**Conclusions:**

Complex and fragmented services struggled to respond holistically to early signs of difficulties and to the young people's cumulative experience of trauma and adversity, mental ill-health and drug-related harms in the context of prohibition. There is a need for a radical rethink of systems to enable integrated youth-centred approaches that meet the needs of those at risk of drug-related deaths and to address the broader social and structural contexts of drug deaths.

## Introduction

Drug-related deaths (DRDs) in Scotland have increased exponentially over the past 20 years and drug death rates have been consistently much higher than in other countries. In 2023, the deaths of 1172 people were registered as “drug misuse deaths” in Scotland: a rate of 22.4 deaths for every 100,000 people.^1^ These rates are more than double those of the other UK nations (NRS, 2024) and are the highest in Europe, albeit there are issues with definitions and under-reporting in some countries (EMCDDA, 2021).

Analyses of DRDs in Scotland and in Europe tend to focus on the drugs implicated in the deaths (EUDA, 2024; NRS, 2024). In Scotland, these are mainly opiates/opioids (such as heroin/morphine and methadone) and benzodiazepines (such as diazepam) both “street” and prescribed (NRS, 2024). In addition, the strong association between socioeconomic position and neighbourhood deprivation with higher levels of drug-related harms (ACMD, 1998 and 2016) is illustrated clearly in Scottish data where people living in the most deprived areas are over 15 times more likely to die from a drug overdose compared to people in the least deprived areas (NRS, 2024) and where absolute inequalities in mortality from drugs have doubled since the beginning of the 2000s (Allik et al., 2020).

Broader contexts of drug deaths have been explored in a growing canon of research, particularly from north America, which has identified a complex relationship between DRDs and a range of socioeconomic factors similar to the social and structural determinants of health inequalities (Marmot, 2005). Such factors include long-term unemployment, economic hardship, low levels of education, neighbourhood decline and spatially concentrated disadvantage; psychosocial issues (such as stigma, stress, poor mental health and physical and psychological trauma); and structural issues (which constrain social welfare and healthcare systems, lead to low treatment coverage and efficacy, housing precarity and increased rates of incarceration) (Altekruse et al., 2020; Bradford & Bradford, 2019; Dasgupta et al., 2018; Galea et al., 2003; Graham, 2020; Heyman et al., 2019; Nosrati et al., 2019; van Amsterdam et al., 2021; Venkataramani & Tsai, 2020).

Further studies from the USA have identified a “deaths of despair” explanation for the increase in drug deaths – a loss of hope and opportunity emanating from a process of cumulative and multiple disadvantage over the lifecourse along with a bleak socioeconomic outlook, progressively worsening labour market opportunities, and the breakdown of social support structures – deaths which for the most part were situated within the risk environments of the post-industrial periphery and linked to policies of deindustrialisation (Case & Deaton, 2015; Graham, 2020; McLean, 2016). Similarly, Scottish research has identified a cohort effect for drug-related deaths among young adult males living in deindustrialising regions. This cohort was seen to have been impacted negatively by the neoliberal socioeconomic policies of the 1980s and 1990s which led to a rapid rise in income inequality and unemployment and a similar breakdown in social support structures (Allik et al., 2020; Parkinson et al., 2018).

There has been growing attention also to research on “Adverse Childhood Experiences” (ACEs) (Felitti et al., 1998) as a driver of drug deaths. ACEs research identified that stressful or traumatic experiences that occur during childhood and youth (such as domestic violence; physical or sexual abuse; emotional neglect; parental separation; and living in a household affected by alcohol and drug use, mental illness, suicide or imprisonment) impacts on future health and substance use. However, ACEs research is seen to neglect the strong association between these experiences and a household's socioeconomic position, and decontextualise childhood adversity from broader structural issues that influence substance use problems (Grummitt et al., 2022; Walsh et al., 2019). For example, the impact of fiscal austerity policies, decommissioning and cuts to addiction services (ACMD, 2017).

These upstream policies, the “causes of the causes” (Marmot, 2018) not only shape inequalities in health and death, but also influence access and utilisation of services. Extensive research over time has demonstrated the effectiveness of drug treatment (Degenhardt et al., 2019; Hser et al., 2015; Strang et al., 2020). People receiving medication assisted treatment are less likely to die from an overdose than those who are not (Hamilton & Stevens, 2018; McAuley et al., 2023; Sordo et al., 2017). Long-term stable treatment, across fewer treatment episodes, is associated with improved outcomes, regardless of treatment modality, although repeated episodes of detoxification are associated with poorer outcomes (Teesson et al., 2017). Data showing contact with in-patient and outpatient services, drug and mental health and criminal justice services (including incarceration) prior to a DRD indicate a deterioration in physical and mental health and drug use and suggest lost opportunities for intervention (Hacker et al., 2018).

However, moralistic drug policies (Stevens, 2019) and addiction recovery policies that focus on abstinence goals in lieu of “living well”, and which struggle to accommodate the role drugs play in people's lives affect engagement with services (Fomiatti et al., 2019; Harris & Rhodes, 2013). Dennis (2021) noted “treatment fatalism” among older people who use drugs whose engagement with services was influenced by their feeling unheard, ill-understood and stigmatised. For young people, Linton et al. (2021) noted youth cognitive development, stigma and structural factors (e.g., disinvestment, lack of youth-centred and integrated services) as barriers to engaging in treatment. Improved treatment outcomes are seen to be related to the availability of services (Galea et al., 2003) and “treatment readiness” (Darke et al., 2005; Flynn et al., 2003); and trust, confidentiality and accessibility identified as influential factors in people's willingness to use services (Park et al., 2020).

Overall, the evidence suggests strongly that social, structural and systemic factors are critical to understanding drug-related deaths among adults (MacGregor, 2017). However, the relevance of these factors to DRDs among young people is not well known. Although drug overdose deaths typically occur among males aged 40 or older (EUDA, 2024), they rank among the highest causes of death for young people. For example, in Scotland, drug deaths are the biggest single contributor to mortality in 15–44-year-olds (Miall et al., 2022:50).

**Figure 1.**
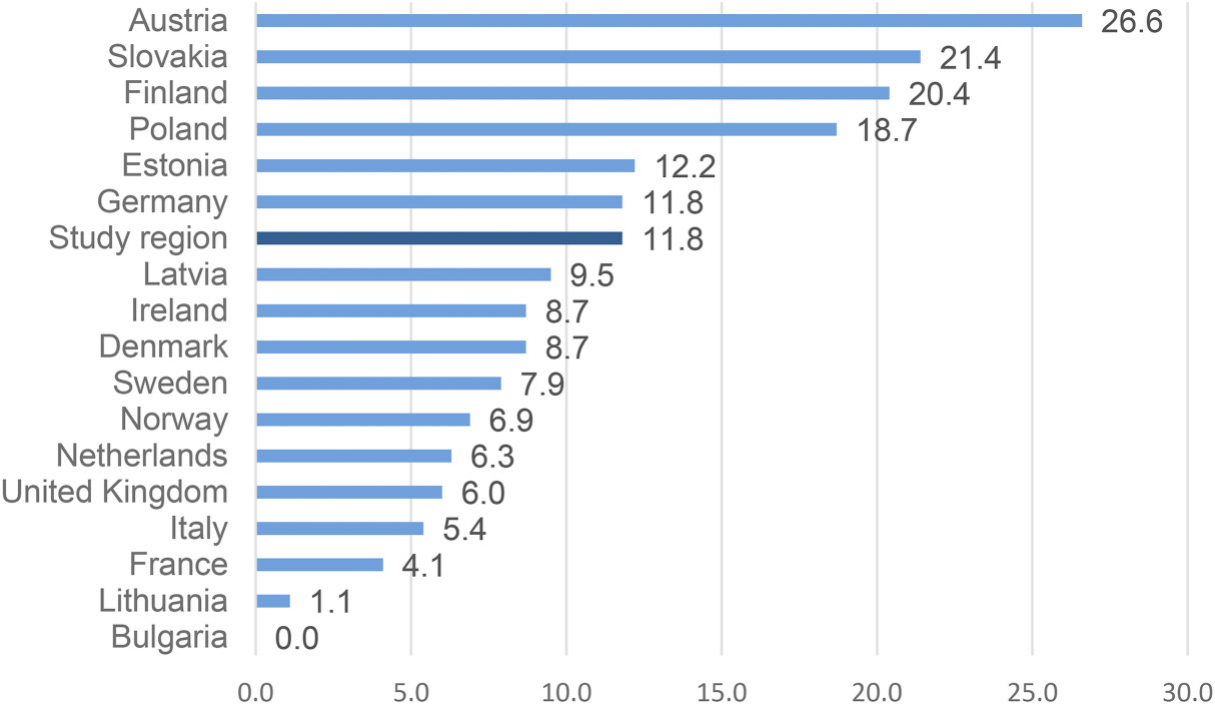
Drug-Induced Deaths (Percentage Under 25 Years) for Selected Countries in the European Union, Norway, UK and the Study Region for 2022 or Most Recent Year Data Available.

Source: EUDA, 2024, National Records Scotland (2024).

In one region of Scotland, the percentage of DRDs that occurred among young people under 25 years of age was twice that of the national (Scottish) figure (12% compared to 6% for the 5-year period 2018–2022). In a European context, the proportion of young DRDs in the study area (12%) are less than in Finland (20%) but considerably higher than Sweden (8%), Norway (7%), the UK as a whole (6%) and many other European countries (Figure 1). This study takes the relatively high cohort of deaths in this Scottish region as a case study to explore young people's life experience, their drug use histories, and their interaction with agencies and services prior to their death with the aim of understanding whether social, structural and systemic factors contribute to young drug deaths as they do adult deaths.

## Methods

The study developed an innovative socio-ecological autopsy approach informed by social autopsy methods and social ecology and risk environment frameworks. This approach enables consideration of the dynamic interaction between people and the broad range of interrelated systems they encounter as well as wider cultural, political and economic contexts and risk environments (Bronfenbrenner, 1977; Rhodes, 2002). Social autopsy research was developed in the 1950s and 1960s to understand the context of maternal and infant deaths in the global south (Kalter et al., 2011). The method was adapted later to understand deaths by suicide (Scourfield et al., 2012) and other public health crises (Klinenberg, 1999). By drawing on this method, our approach sought to look beyond the immediate cause of death and identify broader contextual factors that may have contributed to a death through a “whole-life view” for each young person.

Ethical approval to access confidential information sources was sought and granted for the study by the National Health Service (NHS) Health Research Authority. We choose to describe the region where the research is based as “the study area” (a region of low population density with approximately 300,000 inhabitants spread across mainly rural and small urban communities) to minimise the risk of the young people being identified due to the small numbers involved. For the same reason, care was taken so that our analysis did not reveal, unwittingly, the identity of the young person when the data related to a very small number of people. For this reason, at times we use terms such as most, many and some to protect identities.

Using the National Records of Scotland's definition of drug-related deaths (NRS, 2024) –unintentional (almost all the cases in this study), intentional and undetermined self-poisonings – the deaths of 21 young people, aged 25 years and under, were identified as drug-related. All accessible health, social and criminal justice records (both paper and electronic) from a range of primary and secondary data sources (Table 1) were examined for each person.

**Table 1.** Primary and Secondary Data Sources Available for the Study.

Primary Source Description (Number of Cases)	Records or Reports Accessed
Health records from general hospital services and mental health services (n = 20)	Electronic and paper health records
Health records from specialist drug and alcohol services (n = 13)	Electronic and paper health records
Health records from primary health care (n = 14)	Summary reports from general practices
Police reports relating to the circumstances for each death (n = 21)	Summary reports from Police Scotland
Postmortem and Toxicology reports for each death (n = 21)	Reports provided from Scottish Fatalities Investigation Unit
*Secondary source description*	
National Records of Scotland (NRS) death records (n = 21)	
National Drug-Related Deaths Database (NDRDD) (n = 21)	
Linked Health Dataset from Public Health Scotland (n = 20)	

The quantity and type of records available varied. All known electronic health records such as general medical practitioner (GP) records, letters of referral, hospital appointments and admission records were made available for each person. In addition, we were given access to paper copies of their records, mainly from general and psychiatric hospitals, which had been physically filed in cardboard folders. These files had been collated and archived after the young person's death and were made available for review under controlled access for a limited period of time. Typically, these paper files started with the young person's first hospital appointment and included records (often duplicates of the electronic documents) regarding GP visits, letters of referral to specialist care, physical and mental health assessments, emergency department visits, drug/alcohol services records and hospital admissions. The number and thickness of the files varied depending on their involvement with services, for example there were between one and six paper health files of differing size for each young person. Two of the research team (SG and AO’G) read through the bundles of files in depth and summarised their content in a data collection form which recorded sociodemographic and descriptive data for each young person such as their age, sex, area of residence, official cause of death, references to drug use, mental and physical health, and contact with health, addiction and criminal justice services, along with notes on interventions and professional involvement and contextual information on their lives, their friends and family. All data and reports were imported into NVivo (Lumivero) for coding and thematic analysis (Braun & Clarke, 2021). Excel (Microsoft Corp.) was used to produce descriptive statistics.

### Limitations of the Study

Crucial perspectives are missing from the study, namely the voices of the young people themselves, and the people directly involved in the lives of the young people, both personal and professional. In addition, we had no measure to assess the completeness of the data from each source. For a minority of the young people in this study, there was very little information on their early life and school days, this includes young people who were not born in the study region and/or those who had very little involvement with services. However, in the first ever social autopsy study of this kind, the data collected and analysed revealed sufficient understanding of the trajectory of the lives of young people towards a drug-related death.

## Findings

### Sociodemographic Characteristics of the Young People

Twenty-one deaths of young people were recorded as drug-related in the study area between 2012 and 2019, 18 young men and three young women aged between 16 and 25 years. The median age at death was 22 years. Almost all (n = 18; 86%) were recorded as being of “White Scottish” ethnicity. At the time of their death, nine lived in the region's largest urban area (population approximately 50,000), the remaining in the smaller towns and villages of the area. Two-thirds (n = 14; 67%) lived in the 30% most deprived areas in Scotland (SIND, 2021) (Table 2).

**Table 2.** Number of People in the Study by Sex and Age at Death in Years.

	Sex		
	Female	Male	Total
Number of people	3	18	21
Age at death (years), median	22	22.5	22
Age (years), range	21–23	16–25	16–25

Most of the young people were unemployed at the time of death (n = 12; 57%). A small number (n = 3) were in employment (including community service), two were registered on long term sick leave or with a disability, and the status of the others was unknown. Those who had worked held jobs classified as “routine and manual occupations”, which included work in industries such as construction, hospitality and as tradespeople.

Many of the young people had a peripatetic existence moving frequently as children, teenagers and as young adults: six young people had four to six different addresses and three had nine or more. Accommodation and living arrangements varied over their lifecourse. At the time of death, more than half of the young people (n = 9) were living with relatives, mainly with their mother (n = 5); six people had their own accommodation with two living with their partners; five people were homeless, either living in homeless or supported accommodation and “sofa surfing” with family and friends. Ten of the young people died at their usual place of residence, five people died at their partner's residence and six died either in hospital or in public places.

Three of the young people had children of their own: six children in total. Five were “known to” the children's social work services and four had been removed from their parents’ care. One young person had no contact with their child.

### Drug Use History and Cause of Death

Half of the young people (n = 10; 48%) were identified in their records as intravenous (IV) drug users at the time of their death, and almost half (n = 9; 43%) as using drugs regularly. Alcohol was the most commonly identified drug of use and almost all the cohort (n = 17; 85%) were identified as high-risk drinkers. Polydrug use was common across their lifecourse with a pattern of early onset of alcohol and cannabis use, most commonly in early teens. Later, opioids, benzodiazepines and a range of other substances − stimulants, depressants, empathogens, dissociatives, and over the counter medications – were added to their polydrug repertoire. Almost two-thirds of the young people (n = 13; 63%) had experienced at least one non-fatal overdose (NFOD) prior to their death – a known risk factor for drug-related death. It is likely that this figure is an underestimation as NFOD are not routinely reported.

The main causes of death recorded by the pathologist in the postmortem reports were multi-drug toxicity (n = 13) and heroin toxicity (n = 5). Overall, almost half (n = 10; 48%) of the total deaths involved IV substance use, of which 8 involved heroin. In addition to the cause of death, postmortem reports identified substances implicated in the deaths, mainly heroin (n = 12) and diazepam (n = 10), and a range of additional substances and medications − alcohol, amphetamine, buprenorphine, cannabis, cocaine, codeine, diazepam, methadone, paracetamol and pregabalin among others.

### Physical Health

Twelve of the cases had a number of physical health conditions recorded and almost all of these (n = 10) were receiving treatment from NHS primary and/or secondary care. Conditions, often requiring ongoing care, included asthma, chronic pain, epilepsy, eczema, fractures, persistent headaches, hepatitis C and seizures. At the time of death, three people had no record of being registered with a GP in primary care.

### Mental Health

Almost all of the young people (n = 19; 90%), reported mental health difficulties most commonly related to anxiety, depression and self-harm. References to conditions such as autism spectrum disorder, dissocial personality traits, schizophrenia and social anxiety disorder were also noted. Two-thirds of the cohort (n = 14) received NHS treatment for a mental health concern. Seven received a formal mental health diagnosis – personality disorder (n = 4) and psychosis, psychotic episodes or a drug induced psychosis (n = 3) – and received treatment in Primary Care, Secondary Care and a Community Mental Health Team, along with seven others without a formal diagnosis. A further three young people (one with mental health concerns noted by professionals and three who self-reported mental health concerns) had no record of treatment offered or received.

Eight of the young people had a recorded history of self-harm, and six were known to have attempted suicide previously, five of these young people had a history of both. Similar to the data on NFOD above, it is likely that these figures are an underestimate as self-harm and attempted suicides are not routinely reported. The deaths of five people were suspected to be completed suicides and three of those people were noted to have a history of both self-harm and suicide attempts.

### Early Years

Many of the young people's records contained frequent descriptions of their unhappiness and distress as well as their challenging and aggressive behaviour at home and in school from a young age. Records indicated that almost a quarter of the young people had “behavioural issues” during their school years (n = 5) (including attending school under the influence of drugs and alcohol) and about one-fifth were reported to struggle with learning difficulties (n = 4.) Nearly one-third had poor school attendance (n = 8). During their school years, some of the young people were admitted to hospital for intoxication, while others had contact with the criminal legal system for “anti-social” and violent behaviour. Most of these issues were recorded during their secondary schooling with just a small number raised during primary school.

Within the school setting, measures to support young people ranged from school meetings with parents, a reduction to part-time attendance, and the provision of education in specialist units. Others were excluded from school either temporarily or permanently (n = 5), or families moved them to another school before formal action was taken. There was little evidence of the children and young people being asked about the reasons for their behaviour or their distress, and efforts to understand their perspective appear limited.

In most cases, parents sought or were open to receiving support, such as home visits from social workers, though a small number did not want services to be involved and sought to manage the issues within their family. From the records, we gained an insight into the ways families sought to manage their child's behaviour including arguing with them, reasoning with or disciplining them, and moving them to live with another parent or family member and/or to another area. Many parents and carers did not have the resources to manage challenging behaviours and in some cases had their own struggles with substance use, alcohol and mental health.

### Adverse Life Experiences

**Figure 2.**
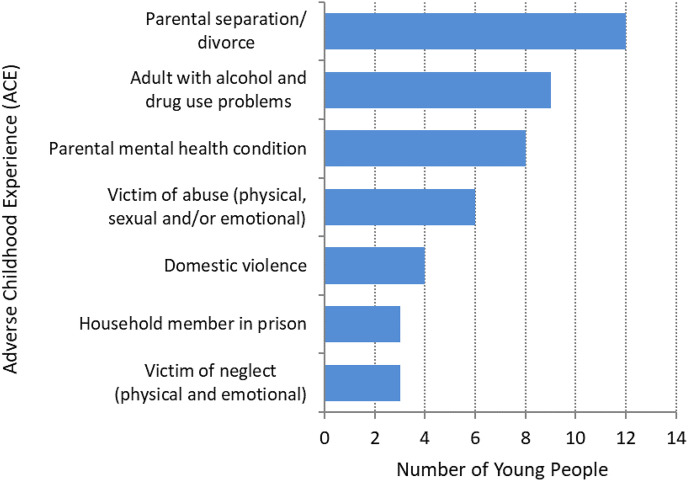
Number of Young People with Adverse Childhood Experience (ACEs) by Type of ACE.

From the records, we identified that almost three quarters of the young people in the study (n = 15; 71%) had a history of ACEs. Six young people had experienced 4 or more adverse events with two experiencing multiple adverse events (8 or 9) during their childhood, the remainder had no ACEs recorded (n = 6). The main adversities the young people experienced included parental separation/divorce (n = 12; 57%), growing up in a household where adults experience alcohol and drug use problems (n = 9; 40%) and having a parent with a mental health condition (n = 8; 38%); seven young people experienced both of the latter within their home. In addition, notes and assessment records indicated that many of the young people experienced, and witnessed, the death of a friend and/or loved one (Figure 2).

One-third of the young people (n = 7; 33%) had been subject to care proceedings or placed on the child protection register as a result of childhood maltreatment (including neglect and physical, sexual and/or emotional abuse) and/or domestic violence within the home combined with alcohol and drug use problems. Almost another one-third of the young people (n = 6; 29%) had similar adverse early childhood experiences but lived away from their parents with extended family.

In addition to experiencing adverse life events in childhood, many of the young people experienced adverse events in the year before their death, such as the breakdown of a significant relationship (n = 5), a health condition (n = 4), a child taken into custody (n = 3), contact with the criminal legal system (e.g., they had been arrested, charged and/or awaiting sentence for an offence) (n = 3), had a close family member who experienced serious ill health and/or disability (n = 2), lost their job (n = 2) and/or had relapsed into more chaotic forms of drug use (n = 2). There were also references in their records to bereavement, financial problems, and homelessness or housing problems prior to their death.

### Contact with the Criminal Legal System

Many young people in the study started using illicit substances regularly in their early teens and within a few years had accumulated debts with some selling drugs to pay for both their drug use and their debts. Contact with the drugs economy and its related violence led to arrests and criminal charges for assault, breach of the peace, theft and drugs offences, and many reported being extremely stressed and anxious about the level of violence in their world. Almost two thirds of the young people (n = 13; 62%) had been violent towards other people and just over half (n = 11; 52%) had been victims of physical assault themselves.

Many of the young people had recent involvement with the prison system − a high-risk factor for drug-related deaths. Over half (n = 11; 52%) had been in prison and almost all had been released within ten months of their death; two had been released less than 2 weeks prior to their death.

### System of Care

Many of the study cohort had contact with statutory services from primary school or their early teens onwards; these included specialist youth services (to support young people who are offending, at risk of offending or have substance use difficulties), “criminal justice social workers” and primary or secondary health care services including child and adolescent mental health services. The more significant interventions were put in place for the children and young people who were “care experienced” and whose behaviour reflected distress from adverse and traumatic experiences within their home.

Most often the first point of help-seeking by the young person, and/or their family, was with their GP. This most often resulted in a written referral sent by the GP to addiction and/or mental health services. There were positive examples of services working across professional disciplines to provide support and reaching out to colleagues for advice on further actions that could be taken to support the young people. However, services were seen to struggle to address young people's behaviours and their complex interconnected needs. Each service had its own approach to young people and each service had its own assessment and plan for what actions were required and what supports were available. In the current fragmented healthcare system (particularly in relation to siloed mental health and drug treatment services), this results in referrals from one practitioner to another. The pattern seen in many of the young people's records is one of multiple referrals and re-referrals from GPs or youth services to specialist mental health and drug treatment services because of the complexity of the problems being described. However, referrals were not always accepted and, instead, young people were signposted elsewhere and/or were encouraged to refer themselves to drug treatment and third sector services. Despite many of the young people in the study using alcohol and drugs from their early teens, which progressed quickly to problematic use, they were not referred to services until they were at least 16 years old.

### Drug Treatment Services

Records showed that almost two thirds of the young people (n = 13; 62%) were referred to “drug and alcohol recovery services” and four were treated there; a further two were referred and attended a residential rehabilitation service. Six of the cohort underwent at least one episode of drug detoxification treatment. There were no records of referral for six young people.

Typically, drug treatment services carry out an assessment, provide a treatment plan and agree a contract of engagement with the young person. At the time of the study, same day assessment or treatment was not available (this is policy now) and opiate agonist treatment (buprenorphine or methadone) usually commenced a few weeks after initial engagement with a focus on abstinence recovery. This was delivered in a shared care system between the addiction services (typically weekly appointments) and the community pharmacy (typically daily appointments).

From the records of the young people who received a referral to treatment, we observed that they there were numerous stages to the assessment process, and many dropped out of the process at an early stage. The emphasis on motivation to change as a condition of treatment was difficult for the young people who were often in crisis by the time they received a referral. Those who did succeed in accessing treatment found it difficult to maintain abstinence from other substances during (their abstinence focussed) treatment and to attend frequent appointments consistently over a sustained period. As a result, “relapse” and missed appointments were frequent. Their files included numerous “Did Not Attend” letters posted to the young people, along with records of attempts to call their mobile phones, to rearrange appointments and outline the consequences of non-attendance. Often the young person did not respond, or did so erratically, which resulted in their discharge from treatment. Subsequent self or GP referrals to treatment services required the assessment process to restart from the beginning. At the time of their death, just two of the young people were receiving opiate agonist treatment.

## Discussion

The present study is the first of its kind to use social autopsy methods and social ecology frameworks to examine drug-related deaths. Our aim was to look at the context of young people's deaths, their drug using histories, their life experience, and their interaction with agencies and services, with the aim of informing policy and practice and preventing further deaths. The study found that, in their short lives, most of this cohort of young people experienced mental and physical ill health, as well as multiple adversities in childhood and as young adults, and we identified five key areas that warrant interventions.

### Early Warnings

In most cases, there were early warning signs of unhappiness and distress among the young people while they were of school going age which manifested in challenging behaviours. However, there appeared to be few resources available to schools and parents to support these young people and intervene positively to effect change. Responses recorded in the files focussed on sanctions, school suspensions and exclusions. In some cases, where maltreatment and abuse were identified at home, children were moved from their family home and placed in formal state or informal kinship care. These interventions did not work for these young people and new thinking is required. As Whittaker et al. (2022, 2023) note, the focus of state interventions on child protection and the governance of “risky” parents serves to marginalise and stigmatise families further and hide alternative approaches to understanding and responding to the complex needs of children and families.

### Precarity and Adversity

Many of this cohort of young people experienced precarious social and economic conditions throughout their short lives − poverty, poor quality and insecure housing, educational disadvantage, unemployment, social exclusion and isolation, violence and imprisonment. Many had a nomadic youth moving from place to place with family members and again as young adults seeking stable and safe accommodation. Almost all experienced adverse and traumatic life circumstances growing up with a parent with substance use and/or mental health problems; and suffered a culmination of adversities in the year preceding their death. Life experiences that are well-established risk factors for problem drug use and overdose (Hser et al., 2015; Lyons et al., 2019; Marel et al., 2024; Park et al., 2020; Peters et al., 2020).

### Systems and Structures

Dasgupta et al. (2018) and Galea et al. (2003), among others, have identified systemic as well as structural issues contributing to harms among people at risk of overdose and a drug-related deaths. Almost all the cohort had poor physical and mental health including high levels of anxiety and distress manifesting in self-harm, overdoses and/or attempted suicide. However, the system of care in place at the time of the study was hard to reach, assessment heavy, and struggled to address their complex and multiple needs. An archaic and often dysfunctional referral system could only accept referrals for one aspect of a young person's difficulties, such as mental health or substance use but not both together, and we observed many instances of staff exasperation at the inflexible and circular referral systems. Navigating this labyrinth of care with different referral criteria for different services was confusing and difficult for the young people, their friends and families, and the professionals tasked with their care. Referrals often focussed on establishing a clinical diagnosis of a mental disorder to enable psychiatric treatment, but this was stymied by the system's incapacity to respond to their ongoing substance use. Referrals for support were made at a relatively late stage, given the early onset of difficulties and a mismatch between needs and services was observable. This suggests insufficient understanding of the severity of these young people's difficulties when they present to services and insufficient under-resourced youth-centred services to meet their needs.

### Recovery?

Drug treatment services required the young people to opt in and demonstrate a motivation for change and agree to, and adhere to, an abstinence-oriented recovery plan. However, most of the young people found this difficult in the context of their adverse and precarious life circumstances and their perception that substances helped them to cope with anxiety and distress. Recovery focussed services struggled to be flexible and support young prople through cycles of using and abstaining, and when their treatment plan failed to translate to real clinical improvement they responded with sanctions rather than supports. At this point, the young people disengaged and/or were discharged from treatment arguably at a time when low-threshold harm reduction and person-centred wraparound services were most needed. In their stead, they were frequent attenders in hospital Emergency Departments for treatment of injuries, mental health episodes and overdoses.

### Criminal Justice

Throughout their young lives, almost all the young people had contact with the criminal legal system and more than half had experienced incarceration. Frequent use of a range of illicit substances brought people into contact with the drugs economy as buyers and/or sellers of illegal goods. Contact with a prohibited industry that uses violence to control its trade (Goldstein, 1985; Windle et al., 2020) led many to become both perpetrators and victims of assault and be over-policed but under-protected. Unlike education, health and social care services where “poor behaviour” and “poor attendance” is sanctioned with the withdrawal of services, the criminal legal system intensifies its contact as behaviour deteriorates and social norms are transgressed. As Duke et al. (2023) note, there is a need for a fundamental rethink as to who should receive a sentence of imprisonment and who could be diverted out of custodial settings into community-based penalties and treatment.

## Conclusions

This study raises critical questions on how best to respond to young people at risk of drug-related harms and deaths. New vision is needed to address the complex and fragmented system of care that results in unmet needs, untreated trauma and mental health conditions, and inappropriate or inaccessible treatment for substance use. Calls for integrated and holistic systems of care have long been made in the UK and elsewhere: yet, there is little evidence of progress despite elevated levels of need. There is a need for spaces that are safe and non-judgemental and committed to ongoing engagement and harm reduction in low-threshold services for those most at risk. This is difficult to to acheive within the context of drug prohibition. There is a further need to addresses the “causes of the causes” that shape health inequalities and the social, structural and systemic determinants of drug-related harms and deaths. Political economic policies that exacerbate inequality, such as austerity and the restructuring and retrenchment of healthcare and the welfare state, results in vulnerable people, such as those at risk of a drug death, disproportionately experiencing policy-induced harms and structural violence (i.e., the avoidable impairment of fundamental human needs (Rylko-Bauer & Farmer, 2016; Galtung, 1990). As a result, addressing drug-deaths requires new thinking about how the distribution of power and resources in society can be shifted to achieve an equilibrium of needs and care.

## Notes

1. The definition of drug-deaths used in Scotland includes all deaths where the underlying cause was drug abuse OR where the underlying cause was poisoning AND there was a controlled substance in the body. This definition excludes cases where drug use indirectly led to the death or where chronic health conditions caused by drug use caused the death. (NRS, 2024).

## References

[bibr1-14550725251370436] ACMD (Advisory Council on the Misuse of Drugs) . (1998). Drug Misuse and the Environment. The Stationery Office.

[bibr2-14550725251370436] ACMD (Advisory Council on the Misuse of Drugs) . (2016). Reducing opioid-related deaths in the UK. https://assets.publishing.service.gov.uk/media/5a801ba3ed915d74e33f87d4/ACMD-Drug-Related-Deaths-Report-161212.pdf

[bibr3-14550725251370436] ACMD (Advisory Council on the Misuse of Drugs) . (2017). Commissioning impact on drug treatment. https://www.gov.uk/government/publications/commissioning-impact-on-drug-

[bibr4-14550725251370436] Allik M. Brown D., Dundas R. , et al. (2020). Deaths of despair: Cause-specific mortality and socioeconomic inequalities in cause-specific mortality among young men in Scotland. International Journal for Equity in Health, 19(215), 10.1186/s12939-020-01329-7 PMC7716282 33276793

[bibr5-14550725251370436] Altekruse S. F. Cosgrove C. M. Altekruse W. C. Jenkins R. A. Blanco C. (2020). Socioeconomic risk factors for fatal opioid overdoses in the United States: Findings from the Mortality Disparities in American Communities Study. PLOS ONE, 15(1), 1–16 10.1371/journal.pone.0227966 PMC6968850 31951640

[bibr6-14550725251370436] Bradford A. Bradford W. (2019). The effect of evictions on accidental drug and alcohol mortality. Health Services Research, 55(1), 9–17 10.1111/1475-6773.13256 31889303 PMC6980953

[bibr7-14550725251370436] Braun V. Clarke V. (2021). Thematic Analysis: A Practical Guide. Sage.

[bibr8-14550725251370436] Bronfenbrenner U. (1977). Toward an experimental ecology of human development. American Psychologist, 32(7), 513. 10.1037/0003-066X.32.7.513

[bibr9-14550725251370436] Case A. Deaton A. (2015). Rising morbidity and mortality in midlife among white non-hispanic Americans in the 21st century. Proceedings of the National Academy of Sciences, 112(49), 15078–15083. 10.1073/pnas.1518393112 PMC4679063 26575631

[bibr10-14550725251370436] Darke S. Ross J. Teesson M. Ali R. Cooke R. Ritter A. Lynskey M. (2005). Factors associated with 12 months continuous heroin abstinence: Findings from the Australian Treatment Outcome Study (ATOS). Journal of Substance Abuse Treatment, 28(3), 255–263. 10.1016/J.JSAT.2005.01.006 15857726

[bibr11-14550725251370436] Dasgupta N. Beletsky L. Ciccarone D. (2018). Opioid crisis: No easy fix to its social and economic determinants. AJPH, 108(2), 182–186. 10.2105/AJPH.2017.304187 PMC5846593 29267060

[bibr12-14550725251370436] Degenhardt L. Grebely J. Stone J. Hickman M. Vickerman P. Marshall B. D. L. Bruneau J. Altice F. L. Henderson G. Rahimi-Movaghar A. Larney S. (2019). Global patterns of opioid use and dependence: Harms to populations, interventions, and future action. The Lancet, 394(10208), 1560–1579. 10.1016/S0140-6736(19)32229-9 PMC7068135 31657732

[bibr13-14550725251370436] Dennis F. (2021). Drug fatalities and treatment fatalism: Complicating the ageing cohort theory. Sociol Health Illn., 43(5), 1175–1190. 10.1111/1467-9566.13278 33955586 PMC7611256

[bibr14-14550725251370436] Duke K. Gleeson H. MacGregor S. Thom B. (2023). The risk matrix: Drug-related deaths in prisons in England and Wales, 2015-2020. Journal of Community Psychology, 52(8), 1056–1077. 10.1002/jcop.22989 36601729

[bibr15-14550725251370436] EMCDDA (European Centre for drugs and Drugs Addiction) . (2021). European Drug Report 2021: Trends and Developments. https://www.euda.europa.eu/publications/edr/trends-developments/2021_en [accessed July 2024]

[bibr16-14550725251370436] EUDA (European Union Drugs Agency) . (2024) European Drug Report 2024: Trends and Developments*.* https://www.euda.europa.eu/publications/european-drug-report/2024_en [accessed July 2024]

[bibr17-14550725251370436] Felitti V. J. Anda R. F. Nordenberg D. Williamson D. F. Spitz A. M. Edwards V. Koss M. P. Marks J. S. (1998). Relationship of childhood abuse and household dysfunction to many of the leading causes of death in adults: The adverse childhood experiences (ACE) study. American Journal of Preventive Medicine, 14(4), 245–258. 10.1016/S0749-3797(98)00017-8 9635069

[bibr18-14550725251370436] Flynn P. M. Joe G. W. Broome K. M. Simpson D. D. Brown B. S. (2003). Recovery from opioid addiction in DATOS. Journal of Substance Abuse Treatment, 25(3), 177–186. 10.1016/S0740-5472(03)00125-9 14670523

[bibr19-14550725251370436] Fomiatti R. Moore D. Fraser S. (2019). The improvable self: Enacting model citizenship and sociality in research on ‘new recovery’. Addiction Research & Theory, 27(6), 527–538. 10.1080/16066359.2018.1544624

[bibr20-14550725251370436] Galea S. Ahern J. Vlahov D. Coffin P. O. Fuller C. Leon A. C. (2003). Income distribution and risk of fatal drug overdose in New York city neighborhoods. Drug and Alcohol Dependence, 70(2), 139–148. 10.1016/S0376-8716(02)00342-3 12732407

[bibr20a-14550725251370436] Galtung J . (1990). Cultural violence. Journal of Peace Research, 27(3), 291–305. 10.1177/0022343390027003005

[bibr21-14550725251370436] Goldstein P. J. (1985). The drugs/violence nexus: A tripartite conceptual framework. Journal of Drug Issues, 15(4), 493–506. 10.1177/002204268501500406

[bibr22-14550725251370436] Graham C. (2020). The role of despair in the opioid crisis: Lessons from the science of well-being. Brookings Institution series on The Opioid Crisis in America (Foreign Policy and Global Economy & Development programs at Brookings), June. https://www.brookings.edu/wp-content/uploads/2020/06/2_Graham_final.pdf

[bibr23-14550725251370436] Grummitt L. Barrett E. Kelly E. Newton N. (2022). An Umbrella review of the links between adverse childhood experiences and substance misuse: What, why, and where do we go from here? Substance Abuse and Rehabilitation, 13, 83–100. 10.2147/SAR.S341818 36411791 PMC9675346

[bibr24-14550725251370436] Hacker K. Davis Jones L. Brink L. Wilson A. Cherna M. Dalton E. Hulsey E. G. (2018). Linking opioid-overdose data to human services and criminal justice data: Opportunities for intervention. Public Health Reports, 133(6), 658–666. 10.1177/0033354918803938 30300555 PMC6225885

[bibr25-14550725251370436] Hamilton I. Stevens A. (2018). Drug deaths increase as fewer people access treatment. The Conversation. https://theconversation.com/record-level-of-drug-deaths-in-england-and-wales-latest-official-figures-99710. Accessed 1 Feb 2018.

[bibr26-14550725251370436] Harris M. Rhodes T. (2013). Methadone diversion as a protective strategy: The harm reduction potential of ‘generous constraints’. Int J Drug Policy., 24(6), e43–e50. 10.1016/j.drugpo.2012.10.003 23199896

[bibr27-14550725251370436] Heyman G. M. McVicar N. Brownell H. (2019). Evidence that social-economic factors play an important role in drug overdose deaths. Int J Drug Policy., 74, 274–284. 10.1016/j.drugpo.2019.07.026 31471008

[bibr28-14550725251370436] Hser Y.-I. Evans E. Grella C. Ling W. Anglin D. (2015). Long-term course of opioid addiction. Harvard Review of Psychiatry, 23(2), 76–89. 10.1097/HRP.0000000000000052 25747921

[bibr29-14550725251370436] Kalter H. D. Salgado R. Babille M. Koffi A. K. Black R. E. (2011). Social autopsy for maternal and child deaths: a comprehensive literature review to examine the concept and the development of the method. Popul Health Metrics, 9(45), 1–13. 10.1186/1478-7954-9-45 PMC3160938 21819605

[bibr30-14550725251370436] Klinenberg E. (1999). Denaturalizing disaster: A social autopsy of the 1995 Chicago heat wave. Theory and Society, 28(2), 239–295. http://www.jstor.org/stable/3108472 https://doi.org/10.1023/A:1006995507723

[bibr31-14550725251370436] Linton S. L. Winiker A. Tormohlen K. N. Schneider K. E. McLain G. Sherman S. G. Johnson R. M. (2021). People don't just start shooting heroin on their 18^th^ birthday": A qualitative study of community Stakeholders’ perspectives on adolescent opioid use and opportunities for intervention in Baltimore, Maryland. Prev Sci., 22(5), 621–632. 10.1007/s11121-021-01226-7 33826057 PMC8024438

[bibr32-14550725251370436] Lyons R. M. Yule A. M. Schiff D. Bagley S. M. Wilens T. E. (2019). Risk factors for drug overdose in young people: A systematic review of the literature. Journal of Child and Adolescent Psychopharmacology, 29(7), 487–497. 10.1089/cap.2019.0013 31246496 PMC6727478

[bibr33-14550725251370436] MacGregor S. (2017). The Politics of Drugs: Perceptions, Power and Policies. Palgrave MacMillan.

[bibr34-14550725251370436] Marel C. Afzali M. H. Sunderland M. , et al. (2024). Predicting Risk of Heroin Overdose, Remission, Use, and Mortality Using Ensemble Learning Methods in a Cohort of People with Heroin Dependence. Int J Ment Health Addiction. Advance online publication. 10.1007/s11469-024-01257-5

[bibr35-14550725251370436] Marmot M. (2005). Social determinants of health inequalities. The Lancet, 365(9464), 1099–1104. March 19. 10.1016/S0140-6736(05)71146-6 15781105

[bibr36-14550725251370436] Marmot M. (2018). Inclusion health: Addressing the causes of the causes. The Lancet, 391(10117), 186–188. 10.1016/S0140-6736(17)32848-9 29137870

[bibr37-14550725251370436] McAuley A. Fraser R. Glancy M. Yeung A. Jones H. E. Vickerman P. Fraser H. Allen L. McDonald S. A. Stone J. Liddell D. Barnstable L. Priyadarshi S. Markoulidakis A. Hickman M. Hutchinson S. J. (2023). Mortality among individuals prescribed opioid-agonist therapy in Scotland, 2011-2020: A national retrospective cohort study. Lancet Public Health, 8(7), e484–e493. 10.1016/S2468-2667(23)00082-8 37295452

[bibr38-14550725251370436] McLean K. (2016) There’s nothing here: Deindustrialization as risk environment for overdose. International Journal of Drug Policy, 29, 19–26. 10.1016/j.drugpo.2016.01.009 26868674

[bibr39-14550725251370436] Miall N. Fergie G. Pearce A. (2022) Health Inequalities in Scotland: Trends in deaths, health and wellbeing, health behaviours, and health services since 2000. Project Report. University of Glasgow. 10.36399/gla.pubs.282637

[bibr40-14550725251370436] Nosrati E. Kang-Brown J. Ash M. McKee M. Marmot M. King L. P. (2019). Economic decline, incarceration, and mortality from drug use disorders in the USA between 1983 and 2014: An observational analysis. Lancet Public Health, 4, e326–e333. 10.1016/S2468-2667(19)30104-5 31279417

[bibr41-14550725251370436] NRS (National Records of Scotland) . (2024). Drug Related Deaths in Scotland, 2023. https://www.nrscotland.gov.uk/statistics-and-data/statistics/statistics-by-theme/vital-events/deaths/drug-related-deaths-in-scotland

[bibr42-14550725251370436] Park J. N. Rouhani S. Beletsky L. Vincent L. Saloner B. Sherman S. G. (2020). Situating the Continuum of overdose risk in the social determinants of health: A new conceptual framework. Milbank Q., 98(3), 700–746. 10.1111/1468-0009.12470 32808709 PMC7482387

[bibr43-14550725251370436] Parkinson J. Minton J. Lewsey J. Bouttell J. McCartney G. (2018). Drug-related deaths in Scotland 1979–2013: Evidence of a vulnerable cohort of young men living in deprived areas. BMC Public Health, 18(357). 10.1186/s12889-018-5267-2 PMC5870372 29580222

[bibr44-14550725251370436] Peters D. J. Monnat S. M. Hochstetler A. L. Berg M. T. (2020). The opioid hydra: Understanding overdose mortality epidemics and syndemics across the rural-urban Continuum. Rural Sociology, 85, 589–622. 10.1111/ruso.12307 PMC8018687 33814639

[bibr45-14550725251370436] Rhodes T. (2002). The ‘risk environment’: A framework for understanding and reducing drug-related harm. The International Journal on Drug Policy, 13(2), 85–94. 10.1016/S0955-3959(02)00007-5

[bibr46-14550725251370436] Rylko-Bauer B. Farmer P. (2016). Structural violence, poverty, and social suffering. In Brady D Burton L. M (Eds.), The Oxford handbook of the social science of poverty. 47–74. Oxford University Press.

[bibr47-14550725251370436] Scourfield J. Fincham B. Langer S. Shiner M. (2012). Sociological autopsy: An integrated approach to the study of suicide in men. Soc Sci Med., 74(4), 466–473. 10.1016/j.socscimed.2010.01.054 20646811

[bibr48-14550725251370436] SIMD (Scottish Index of Multiple Deprivation) . (2021). https://www.gov.scot/collections/scottish-index-of-multiple-deprivation-2020 [accessed May 2021]

[bibr49-14550725251370436] Sordo L. Barrio G. Bravo M.J. Indave B.I. Degenhardt L. Wiessing L. Ferri M. Pastor-Barriuso R. , (2017). Mortality risk during and after opioid substitution treatment: Systematic review and meta-analysis of cohort studies. BMJ*.* 357, j1550. 10.1136/bmj.j1550 PMC5421454 28446428

[bibr50-14550725251370436] Stevens A. (2019). Being human’ and the ‘moral sidestep’ in drug policy: Explaining government inaction on opioid-related deaths in the UK. Addictive Behaviors, 90(March), 444–450. 10.1016/j.addbeh.2018.08.036 30220439

[bibr51-14550725251370436] Strang J. Volkow N. D. Degenhardt L. , et al. (2020). Opioid use disorder. Nature Reviews Disease Primers, 6(3). 10.1038/s41572-019-0137-5 31919349

[bibr52-14550725251370436] Teesson M. Marel C. Darke S. Ross J. Slade T. Burns L. Lynskey M. Memedovic S. White J. Mills K. L. (2017). Trajectories of heroin use: 10–11-year findings from the Australian treatment outcome study. Addiction, 112(6), 1056–1068. 10.1111/add.13747 28060437

[bibr53-14550725251370436] van Amsterdam J. van den Brink W. Pierce M. (2021). Explaining the differences in opioid overdose deaths between Scotland and England/Wales: Implications for European opioid policies. European Addiction Research, 27(6), 399–412. 10.1159/000516165 33965949 PMC8686715

[bibr54-14550725251370436] Venkataramani A. S. Tsai A. C. (2020). Housing, housing policy, and deaths of despair. Health Serv Res., 55(1), 5–8. 10.1111/1475-6773.13260 PMC6980955 31981232

[bibr55-14550725251370436] Walsh D. McCartney G. Smith M ,. et al. (2019) Relationship between childhood socioeconomic position and adverse childhood experiences (ACEs): A systematic review. Journal of Epidemiology & Community Health, 73*:* 1087–1093. 10.1136/jech-2019-212738 31563897 PMC6872440

[bibr56-14550725251370436] Whittaker A ,. et al. (2023). Family Stories: Lessons for Practice. Preliminary findings from Governing parental opioid use: a relational ethnography. https://relations.stir.ac.uk/

[bibr57-14550725251370436] Whittaker A. Elliott L. Taylor J. Dawe S. Harnett P. Stoddart A. Littlewood P. Robertson R. Farquharson B. Strachan H. (2022). The parents under pressure parenting programme for families with fathers receiving treatment for opioid dependency: The PuP4Dads feasibility study. Public Health Research, 10(3), 1–153. 10.3310/YOWK7214 35129937

[bibr58-14550725251370436] Windle J. Moyle L. Coomber R. (2020). “Vulnerable” kids going country: Children and young people’s involvement in county lines drug dealing. Youth Justice, 20(1-2), 64–78. 10.1177/1473225420902840

